# Ionizing Radiation-Induced Responses in Human Cells with Differing *TP53* Status

**DOI:** 10.3390/ijms141122409

**Published:** 2013-11-13

**Authors:** Razmik Mirzayans, Bonnie Andrais, April Scott, Ying W. Wang, David Murray

**Affiliations:** Department of Oncology, University of Alberta, Cross Cancer Institute, Edmonton, AB T6G 1Z2, Canada; E-Mails: bonnie.andrais@albertahealthservices.ca (B.A.); april.scott@albertahealthservices.ca (A.S.); ywwang@ualberta.ca (Y.W.W.); david.murray5@albertahealthservices.ca (D.M.)

**Keywords:** ionizing radiation, p53, WIP1, premature senescence, apoptosis, endopolyploidy

## Abstract

Ionizing radiation triggers diverse responses in human cells encompassing apoptosis, necrosis, stress-induced premature senescence (SIPS), autophagy, and endopolyploidy (e.g., multinucleation). Most of these responses result in loss of colony-forming ability in the clonogenic survival assay. However, not all modes of so-called clonogenic cell “death” are necessarily advantageous for therapeutic outcome in cancer radiotherapy. For example, the crosstalk between SIPS and autophagy is considered to influence the capacity of the tumor cells to maintain a prolonged state of growth inhibition that unfortunately can be succeeded by tumor regrowth and disease recurrence. Likewise, endopolyploid giant cells are able to segregate into near diploid descendants that continue mitotic activities. Herein we review the current knowledge on the roles that the p53 and p21^WAF1^ tumor suppressors play in determining the fate of human fibroblasts (normal and Li-Fraumeni syndrome) and solid tumor-derived cells after exposure to ionizing radiation. In addition, we discuss the important role of WIP1, a p53-regulated oncogene, in the temporal regulation of the DNA damage response and its contribution to p53 dynamics post-irradiation. This article highlights the complexity of the DNA damage response and provides an impetus for rethinking the nature of cancer cell resistance to therapeutic agents.

## 1. Introduction

Maintenance of genome integrity and cellular homeostasis in human cells are regulated by an intricate network of pathways that include cell cycle checkpoints, DNA repair and recombination. Under normal conditions, exposure to genotoxic agents results in rapid activation of these pathways which serve to promote cell survival by eliminating genomic injury. Cells that experience a degree of DNA damage that is beyond repair may die (e.g., through apoptosis, necrosis and/or autophagy) [1,2]; they may be triggered to undergo an extended proliferative block [3]; or they may execute aberrant mitosis and give rise to aneuploid offspring, a subset of which may remain viable and exhibit extended life span [4].

The wild-type p53 tumor suppressor, encoded by the *TP53* gene, functions at the hub of the DNA damage surveillance network that restricts aberrant cell growth in response to genotoxic stress [5–7]. This network restricts cellular growth by inducing genetically-regulated responses such as apoptosis or stress-induced premature senescence (SIPS), depending on the type of genotoxic insult and the genetic background of the cells [8–10]. Apoptosis, a form of programmed cell death, is characterized by membrane blebbing, cytoplasmic shrinkage, condensation of the chromatin, and ultimately cell death [9,11]. It is orchestrated by complex signaling pathways which involve a family of cysteine proteases called caspases, a subset of which (e.g., caspase-3) directly regulate cell death by degrading vital cellular proteins [11]. SIPS, on the other hand, is a growth-arrested state in which the cells acquire flattened and enlarged morphology, express the marker senescence-associated β-galactosidase (SA-β-gal), and cease to synthesize DNA, but remain viable and secrete growth- and tumor-promoting factors [12,13].

In this article, we review the current state of knowledge regarding the responses induced by ionizing radiation in human fibroblasts and solid tumor-derived cells with differing *TP53* status. Specifically, we focus on: (i) activation and temporal regulation of the DNA damage response post-irradiation; (ii) influence of p53 dynamics on cell fate after radiation exposure; (iii) whether p53 signaling positively or negatively regulates apoptosis in response to DNA damage; (iv) unwanted side effects associated with SIPS; and (v) influence of wild-type p53 loss on radiation-induced responses in terms of clonogenic survival, apoptosis, SIPS and genomic instability. This article is meant to be complimentary to (rather than overlapping with) recent review articles on the various aspects of the DNA damage response published by us and others (Table A1), including a review article [14] that was recently published in this special issue on Radiation Toxicity in Cells. Our purpose is to discuss the complexity of the DNA damage response which goes far beyond the integration of classical DNA repair, cell cycle checkpoints, and apoptosis, and to highlight the potential pitfalls when extrapolating results obtained with different cell types and different DNA-damaging agents.

## 2. Activation and Temporal Regulation of the DNA Damage Response Post-Irradiation

**Figure 1:**
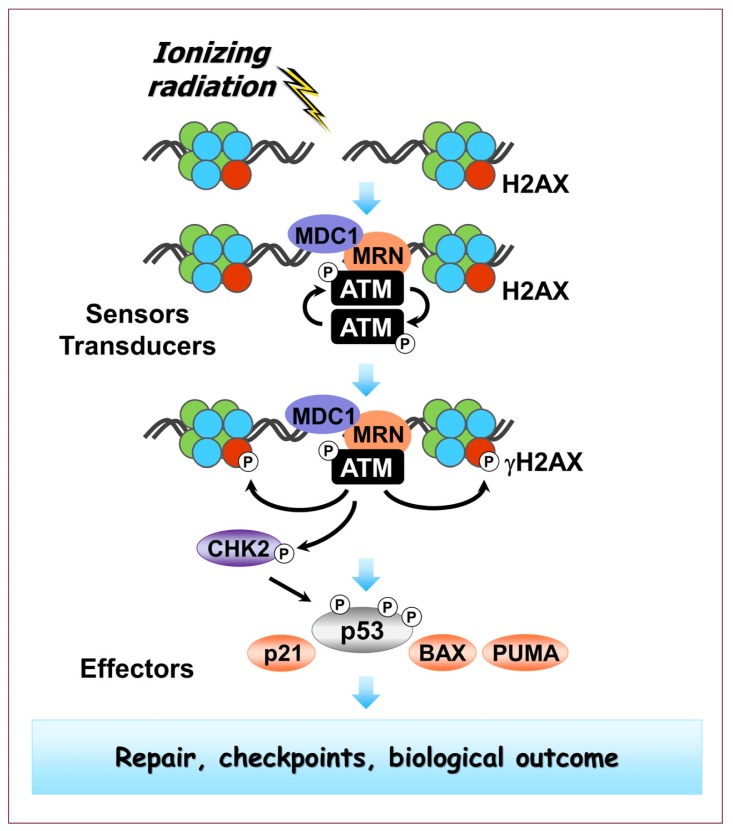
Highly simplified schematic of the radiation-triggered DNA damage response discussed in this article. Open circles containing “P” indicate phosphorylation events. MRN: Mre11-Rad50-Nbs1 complex; MDC1: mediator of DNA damage checkpoint 1, BAX: Bcl-2-associated X protein; PUMA: p53 upregulated modulator of apoptosis.

DNA double-strand breaks (DSBs) are the most deleterious and intensively studied lesions induced by ionizing radiation. Cellular response to DSBs is developed through a series of steps, involving sensor, transducer and effector proteins (Figure 1) (reviewed in [15]). DSBs are first detected by sensors. These might recognize the DNA lesion itself or chromatin alterations caused by DSBs. Next, transducers are recruited to the damage site that serve to assemble the DSB-repair complex at the site of damage and/or activate the downstream signaling, that is, to convey the DSB signal to the effectors. In response to ionizing radiation, the initial and primary transducer is ataxia telangiectasia mutated (ATM), a member of the phosphatidylinositol 3-kinase-related (PI3KK) family of protein kinases. ATM transmits the message via various means, including phosphorylation of proteins such as the histone variant H2AX, p53 and checkpoint kinase 2 (CHK2). Other PI3KK family members, including ATM- and Rad3-related (ATR), also participate in DSB signaling, particularly at late times after irradiation [16,17].

Phosphorylation of H2AX on Ser139 is an important event in the response of mammalian cells to DSBs [18]. Numerous H2AX molecules in the chromatin, surrounding each DSB, are phosphorylated on Ser139 within minutes after irradiation. H2AX molecules that are phosphorylated on Ser139 are referred to as γH2AX. The formation of these γH2AX molecules around each DSB gives rise to “γH2AX foci” which can be visualized by immunofluorescence techniques using Ser139-specific γH2AX antibodies. These γH2AX foci are believed to serve as a platform for the recruitment of DNA repair and checkpoint signaling factors [18,19].

In unstressed cells, p53 is maintained at low steady-state levels to restrict its impact on the cell [20]. Exposure to ionizing radiation triggers post-transcriptional modifications of p53 at multiple sites which contribute to the stabilization of p53 and activation of its biological functions [21]. Of the many different types of modifications on p53, phosphorylation has been the most well-studied both biochemically and genetically. Phosphorylation of p53 on various residues can be mediated not only by ATM and ATR but also by checkpoint kinase 1 (CHK1) and CHK2 [22], p38 mitogen-activated protein kinase (MAPK) [23–25], and other protein kinases [26]. Upon activation, p53 regulates DNA repair, transient cell cycle checkpoints, apoptosis and terminal growth arrest (*i.e.*, SIPS). p53 exerts these effects both directly, through protein-protein interaction (e.g., interacting with key mediators of DSB repair and apoptosis [27]), and indirectly by controlling the transcription of a host of p53-responsive genes [28].

**Figure 2:**
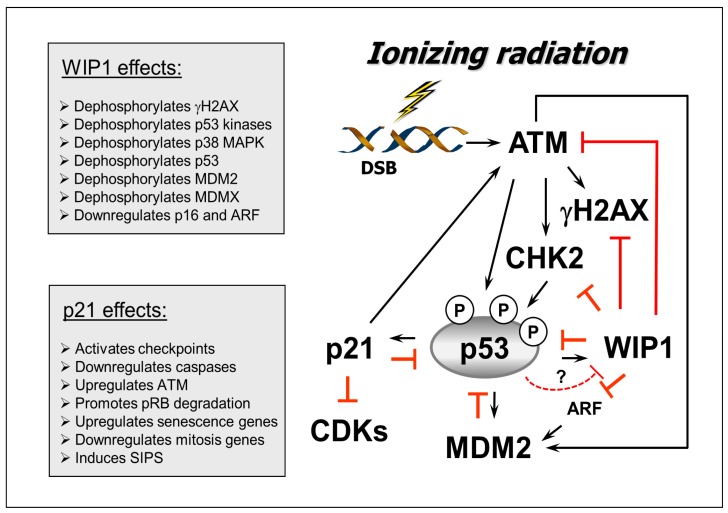
A schematic of the p53-MDM2, p53-WIP1, and p53-p21 regulatory loops discussed in this article. Arrows indicate stimulation and T-shaped lines indicate inhibition. Multiple functions of WIP1 and p21 in the DNA damage response are indicted.

There are a number of excellent reviews covering the structure, regulation, and function of p53 (Table A1). Below, we will focus on MDM2 (murine double minute-2 homologue), WIP1 (wild-type p53-induced phosphatase 1) and p21 in terms of their regulatory interaction with p53 following irradiation. A schematic of these interactions is presented in Figure 2.

### 2.1. The p53-MDM2 Interaction

MDM2 (also known as HDM2 in human) belongs to a large family of RING-finger-containing proteins and functions mainly, if not exclusively, as an E3 ligase [29,30]. It targets p53 for mono- and/or poly-ubiquitylation thereby controlling its localization and/or levels by proteasome-dependent degradation. MDM2-mediated mono-ubiquitylation of p53 results in cytoplasmic sequestration, whereas poly-ubiquitylation triggers p53 degradation. MDM2 can also suppress p53 function by binding to p53, thereby preventing its ability to interact with the basal transcriptional machinery and transcriptional co-activators such as p300 [30–32]. In response to DNA damage, phosphorylation of p53 on Ser20 and of MDM2 on Ser395, mediated by kinases such as ATM, interrupts the p53-MDM2 interaction, resulting in nuclear accumulation of p53 and activation of its transcriptional program.

### 2.2. The p53-WIP1 Interaction

Rapid activation of the DNA damage response machinery post-irradiation followed by repair of genomic injury must be followed by restoration of the cell to its pre-stress state to allow the maintenance of cell homeostasis and resumption of normal growth. This critical function is largely accomplished by WIP1 (also known as PPM1D), a type 2C serine/threonine phosphatase [33]. The human WIP1 was initially identified as a gene whose expression is induced by ionizing radiation in a p53-dependent manner [34]. This gene was subsequently shown to specify a 605 amino acid nuclear protein which can be subdivided into two major domains: a highly conserved *N*-terminal phosphatase domain (amino acids 1–375) and a less conserved non-catalytic domain (amino acids 376–605) which contains two putative nuclear localization signals [33].

Upon transcriptional induction and nuclear accumulation consequent to radiation exposure, WIP1 directly dephosphorylates γH2AX [35–37] which plays a major role in the DNA damage response and maintenance of genomic stability [18,19]. In addition, WIP1 inactivates p53 in several ways. WIP1 directly dephosphorylates p53 at Ser15 [38]. It also mitigates p53 function indirectly, by reducing its phosphorylation at Ser15, Ser20, Ser33 and Ser46 through inactivation of ATM, ATR, CHK1, CHK2, and p38MAPK [38–41]. Another mechanism of WIP1 action on p53 is through influencing the p53-MDM2 interaction [42]. Concomitant phosphorylation of p53 on Ser20 and of MDM2 on Ser395 is critical for interruption of the p53-MDM2 regulatory loop, and hence p53 stabilization. WIP1 restates this negative regulatory interaction not only by inhibiting p53 phosphorylation at Ser20 but also by dephosphorylating MDM2 on Ser395 [42].

WIP1 may regulate p53 signaling not only by influencing p53 protein stability, but also by repressing transcription of p53 target genes. The latter property of WIP1 has been proposed to be mediated by MDMX (also called MDM4 in human). MDMX shares strong homology to MDM2 at the N-terminal domain through which both proteins bind to p53 [43]. The interplay between MDM2 and MDMX in regulating p53 is complex and incompletely understood [44,45]. However, it is well known that, unlike MDM2, MDMX does not have intrinsic E3-ligase activity for p53 but instead has the ability to repress p53 [44]. According to Zhang *et al.* [46], ionizing radiation triggers WIP1-mediated dephosphorylation and stabilization of MDMX, which in turn promotes suppression of the p53 transcriptional program.

The observations made with cultured mammalian cells that WIP1 is capable of interfering with several tumor suppressors were consistent with the notion that this p53-regulated phosphatase might be an oncogene (reviewed in [33]). Studies with various cancer mouse models have validated that WIP1 acts as an oncogene not only by inhibiting tumor suppressors (e.g., p53), but also by complementing other oncogenes (e.g., H-Ras-1 [33]). Furthermore, the genomic region containing the *WIP1* locus (17q23.2) has been reported to be frequently amplified in several human malignancies where *TP53* mutations are less common, including breast cancers, ovarian clear cell adenocarcinomas, neuroblastomas, and pancreatic cancers [47–49].

These properties of WIP1 are intriguing, but its involvement in p53 signalling is rather puzzling. Why would a potent oncogene (WIP1) be positively regulated by a prominent tumor suppressor (p53)? With respect to the DNA damage response, does p53 make the “decision” whether to keep WIP1 under check (to allow repair by p53 and its downstream effectors [27]) or to engage WIP1 (to interrupt and/or shut down the DNA damage response machinery), or is the engagement of WIP1 in this response simply a passive consequence of the sequential execution of these biochemical feedback loops? The following discussion will hopefully provide some insight into these questions.

### 2.3. Multiple Functions of p21

The p21 protein (also called WAF1, CAP20, CIP1, and SDI1) is the founding member of the CIP/KIP family of cyclin-dependent kinase (CDK) inhibitors [50,51]. Although much attention has been directed towards its ability to influence cell cycle progression by inhibiting the activity of cyclin/CDK complexes (e.g., CDK1, 2 and 4), over a decade ago it was established that p21 is a multifunctional protein capable of suppressing apoptosis by mechanisms that cannot be attributed to its cytostatic (cell cycle checkpoint) effects [52,53]. Instead, the anti-apoptotic property of p21 relies on its ability to inhibit cytochrome c release from mitochondria [54], and to inhibit the activity of proteins directly involved in the induction of apoptosis, including caspase 3, caspase 8, caspase 9, caspase 10, stress-activated protein kinases (SAPKs) and apoptosis signal-regulating kinase 1 (ASK1, also called MAP3K5) [54–56]. In addition, p21 suppresses apoptosis by directly controlling transcription, resulting in downregulation of pro-apoptotic genes [56] and upregulation of genes that encode secreted factors with anti-apoptotic activities [55,56].

Another important role of p21 in the p53 pathway is to switch on the growth-arrest (SIPS) program. This property of p21 is ascribed to its ability to inhibit CDKs [57], as well as transcriptionally activating senescence-associated genes coupled with repressing genes involved in mitosis [12,58]. Sustained upregulation of p21 appears to be crucial for the maintenance of the SIPS program consequent to therapeutic exposures [54,59,60]. Interestingly, p21 forms a positive regulatory loop with ATM [59,60] and this interaction facilitates the sustained p21-dependent growth arrest associated with SIPS [59]. This interaction also contributes to the apoptosis-resistant phenotype of cells undergoing SIPS. The latter conclusion is based on the finding that targeting either ATM or p21 in cancer cells that have undergone SIPS triggers death through apoptosis [59].

There is also evidence that p21 may function as a negative regulator of p53. Support for this notion has been provided in part by studies involving the HCT116 human colon carcinoma cell line that expresses wild-type p53 and p21 proteins and isogenic sub-lines derived from this cell line in which either one or both alleles of *p21^WAF1^* are deleted (*i.e.*, HCT116p21+/− and HCT116p21−/−, respectively). The constitutive level of p53 was low in parental HCT116 cells, elevated in HCT116p21−/− cells (~10 fold higher than in parental cells), and intermediate in HCT116p21+/− cells [61]. Javelaud and Besançon [62] demonstrated that p21 deficiency leads to elevated expression of p14^ARF^, which was previously shown to promote p53 stability through binding to its negative regulator, MDM2 [63,64]. Subsequent studies with HCT116 and HT1080 (human fibrosarcoma) cell lines confirmed a role for p21 in negative regulation of p53, but did not implicate MDM2 in this response [65].

## 3. Influence of p53 Dynamics on Cell Fate Post-Irradiation

In 1998, we reported studies with non-cancerous human skin fibroblast strains with differing *TP53* status which led us to propose a “two wave” model for activation of the p53 pathway by DNA-damaging agents [66]. According to our model, while the rapid activation of the p53 pathway (first wave) by ionizing radiation or 254 nm ultraviolet light (hereafter UVC) activates transient cell cycle checkpoints and facilitates DNA repair, the persistence of genetic changes (e.g., DNA damage, chromosome aberrations) provides the critical signal for the late activation of p53 signaling (second wave), resulting in p21-mediated senescence-like growth arrest (now called SIPS) or p53-mediated apoptosis, depending on the cell type and extent of genomic injury. For both agents administered at moderate doses that are typically used in clonogenic survival assays (*i.e.*, 1–8 Gy of ionizing radiation and 1–10 J/m^2^ of UVC), the primary response contributing to loss of colony-forming ability was shown to be SIPS and not apoptosis [66,67–70].

In 2004, Lahav and associates [71] reported their ground-breaking study demonstrating that p53 levels rise and fall in a wavelike or “pulsed” manner in the MCF7 human breast carcinoma cell line following exposure to ionizing radiation. This group identified both MDM2 [71] and WIP1 [72] as major negative regulators of p53 at various p53 waves. These observations provided an impetus for a number of studies, involving both mathematical simulations and biological evaluations, which were designed to uncover the basis for the “digital” p53 response and the biological consequences of different p53 waves.

### 3.1. Mathematical Modeling of p53 Dynamics and Cell Fate Post-Irradiation

Zhang *et al.* [73–75] and Tian *et al.* [76] constructed an integrative computational model of p53 signaling for different doses of ionizing radiation between 1 and 10 Gy. The authors incorporated both the p53-MDM2 and the ATM-p53-WIP1 negative feedback loops, and predicted a “digital” p53 response mechanism, reflecting recurrent p53 initiation pulses triggered by ATM pulses. The integrated model proposed by these authors was composed of a DSB sensor module, a DSB repair module, a p53-centered negative feedback control module, and a cell fate decision module that was limited to G1 “arrest” (presumably the transient activation of the G1/S checkpoint) and apoptosis. The model included two forms of p53 based on its phosphorylation status: p53 “arrestor” that reflects phosphorylation of p53 at Ser15 and Ser20, and p53 “killer” that is further phosphorylated at Ser46. These two forms of p53 were predicted to be separately responsible for inducing G1 “arrest” and apoptosis, respectively. This prediction was based on an earlier report demonstrating that phosphorylation of p53 on different residues including Ser46 is associated with a high degree of apoptosis in MCF7 breast cancer cells subjected to very high levels of DNA damage (*i.e.*, exposure to 50 J/m^2^ of UVC) [77].

The model proposed by these authors predicted that 3 and 7 Gy doses of ionizing radiation would result in ~80% and ~100% apoptosis within ~12 h post-irradiation, respectively [73–76]. The previous observations of others, that mouse thymocytes are highly sensitive to undergoing apoptosis in response to such radiation doses [78–80], was taken as an apparent support for the computational model [76]. However, whether the apoptosis susceptibility of mouse thymocytes is directly linked to the “killer” p53 (Ser46 phosphorylated) remains unknown. With most other cell types, ionizing radiation does trigger phosphorylation of p53 on multiple residues including Ser46, but this effect is not necessarily associated with apoptosis (see below).

Unfortunately, the computational models reported by Zhang *et al.* [73–75], Tian *et al*. [76] and others (e.g., [81]; also see Section 7 below) did not take into account the multiple functions of p21 in the ATM-p53 pathway discussed herein in the context of its sustained upregulation that can persist for several days post-irradiation, particularly its important role in suppressing apoptosis by acting at different levels of the death cascade and its ability to switch on the SIPS response (see above). Thus, while these simulations might predict the consequences of irradiation in cell types such as lymphocytes [82] and thymocytes [78–80] that are prone to undergoing apoptosis after different stimuli, they should not be applicable to cell types such as fibroblasts and p53 wild-type solid tumor-derived cells that predominantly engage p21-mediated SIPS after exposure to moderate doses of ionizing radiation.

### 3.2. Evaluation of p53 Dynamics and Cell Fate in Human Solid Tumor-Derived p53 Wild-Type Cell Lines Post-Irradiation

Batchelor *et al.* [72] determined the dynamics of the global p53 protein levels in MCF7 cells following exposure to a 10-Gy dose of ionizing radiation. They observed two peaks of p53 upregulation over a period of 10 h after irradiation, the first at ~2 h and the second at ~8 h. The initial p53 pulse was linked to repair/checkpoint activation and the late pulse was presumed to be responsible for triggering apoptosis. The cell fate was evaluated in a follow-up study by these authors and shown to be SIPS and not apoptosis [83].

MCF7 cells, which have been extensively used for p53 dynamics [71,72,83] and cell fate studies (e.g., [84–87]), express wild-type p53 but do not express caspase 3 and are relatively insensitive to undergoing apoptosis consequent to therapeutic exposures [88–90]. We determined p53 dynamics and cell fate in A172 human malignant glioma cells which express wild-type p53 and possess a functional apoptotic caspase cascade [7]. We confirmed the occurrence of sequential waves of global p53 upregulation in A172 cells exposed to ionizing radiation (5 Gy), and further demonstrated that p53 upregulation reflects phosphorylation (e.g., at Ser15 and Ser46) and nuclear accumulation of p53 [7]. In addition, we identified a delayed p53 wave, arising several days post-irradiation, that is accompanied by delayed induction of p21 and coincides with the onset of SIPS [7]. Ionizing radiation administered at 5 or 10 Gy does not, however, trigger significant apoptosis in A172 cells [7]. Thus, Ser46-phosphorylated p53 does not function as p53 “killer” in terms of apoptosis in these cells.

In short, it is well established that, in p53 wild-type cells, ionizing radiation triggers sequential waves of p53 signaling that persists for several days post-irradiation, and that the predominant cell fate in many cell backgrounds including epithelial solid tumor-derived cell lines is SIPS and not apoptosis. However, the functional relevance of these sequential waves of p53 induction/activation remains to be elucidated.

## 4. Does p53 Signaling Positively or Negatively Regulate Apoptosis in Response to DNA Damage?

The discovery of the involvement of p53 in the radiation-responsive DNA damage surveillance network in the early 1990’s led to a model in which p53 signaling either promotes survival by activating cell cycle checkpoints to facilitate repair or eliminates injured cells by inducing apoptosis (e.g., [91]). Although this model is still being widely cited and the concepts embodied therein have been important drivers of research in this field, it has long been realized that the biological consequences of ionizing radiation (arrest versus apoptosis) are largely dependent on the genetic makeup of the cells rather than on “decision making” by p53 (e.g., [66,92]). In the early 1990’s, a series of manuscripts published by Kastan and associates ([92] and references therein) led to the conclusion that “*induction of p53 by ionizing radiation leads to a G1 arrest in certain cell types (*e.g., *fibroblasts) and to apoptosis in other cell types (*e.g., *hematopoietic cells). Loss of p53 function would lead to radioresistance in cell types utilizing the apoptosis part of the pathway*.” These observations were made using comparable doses of ionizing radiation (e.g., 4 Gy) for different cell types. Since then, a number of reports [54,93–96], including our own [67,97], have established that SIPS is a predominant response triggered by moderate doses (e.g., between 1 and 8 Gy) of ionizing radiation in most p53 wild-type non-hematopoietic cell types, including human fibroblasts and epithelial cells. In addition, cells undergoing SIPS exhibit apoptosis resistance [54,59,98–100] as a consequence of sustained upregulation of p21 [54,59]. Thus, activation of the p53–p21 axis post-irradiation predominantly triggers SIPS coupled with abrogation of apoptosis in these cell types.

Some reports concluding that activation of p53 by DNA damage can trigger apoptosis have relied on experiments with cells exposed to very high fluences of UVC, usually ranging from 20 to 50 J/m^2^ (e.g., [26,77,101–104]). We refer to such fluences as “supralethal” because exposure of normal human fibroblasts to a much lower fluence (10 J/m^2^) results in a more than 90% decrease in their colony-forming ability [66,68,105,106]. Caution should be exercised in extrapolating results from these experiments because we [66,68] and others [103–110] have demonstrated that moderate and supralethal fluences of UVC trigger different responses in human fibroblasts, predominantly reflecting SIPS and apoptosis, respectively. In studies reported by us [66,68], exposure of fibroblasts to <15 J/m^2^ of UVC resulted in a moderate (~3 fold) up-regulation of p53, prolonged up-regulation and nuclear accumulation of p21, sustained growth arrest, and features of SIPS (flattened and enlarged cell morphology coupled with positive staining for SA-β-gal). Despite causing marked loss of colony-forming ability, such fluences of UVC did not induce cell death (apoptosis or necrosis) at levels detectable by several standard assays that we employed [66,68]. In sharp contrast, exposure to supralethal fluences (e.g., 30 J/m^2^) resulted in a striking (~20 fold) up-regulation of p53, inhibition of p21 expression, prolonged suppression of global RNA synthesis, and a high degree of apoptotic cell death [66,68]. We have now extended these studies to p53 wild-type solid tumor-derived cell lines, and demonstrated that UVC triggers a significant degree of apoptosis only when administered at very high fluences (>20 J/m^2^) (unpublished observations).

The finding that exposure to very high fluences of UVC triggers striking upregulation of p53 accompanied by extensive apoptosis [66,68] is not surprising. Unlike DNA lesions induced by ionizing radiation, UVC induces bulky DNA lesions, referred to as UV photoproducts, the presence of which generates a physical blockage for the transcription machinery [108,110]. Photoproducts induced by moderate fluences of UVC are rapidly removed from expressed genes by the transcription-coupled sub-pathway of nucleotide excision repair [111]. Thus, moderate fluences of UVC trigger a response in p53 wild-type human cells similar to that induced by ionizing radiation [66,68,106], that is, activation of the p53 transcriptional program encompassing p21-mediated downregulation of apoptosis and induction of SIPS. Supralethal fluences of UVC (e.g., 30 J/m^2^), on the other hand, induce levels of photoproducts in expressed genes that overwhelm the excision repair machinery [66]. As a consequence of these persistent photoproducts blocking the transcriptional activation of p21 (anti-apoptotic) and negative regulators of p53 (e.g., MDM2), very high fluences of UVC (>15 J/m^2^) trigger marked upregulation of p53 coupled with a high degree of apoptosis [66].

We proposed the above model for non-cancerous human fibroblast strains over a decade ago [66]. Whether our model is applicable to other cell types and all expressed genes remains largely unknown. Intriguingly, however, consistent with our model, Xia *et al.* [104] recently reported that exposure of the MCF7 breast carcinoma cell line to a high fluence of UVC (50 J/m^2^) resulted in marked upregulation of p53 coupled with suppression of not only p21, but also MDM2 and WIP1. It is noteworthy that this fluence of UVC induced PARP cleavage (a molecular marker of apoptosis) and suppression of cyclin D1 and cyclin B1, but led to an increase in cyclin E and GADD45α protein levels [104]. It remains to be determined whether UVC-induced bulky lesions might be removed more efficiently from some expressed genes (e.g., *GADD45α*) than others (e.g., *WIP1*) in certain cell types.

## 5. Unwanted Side Effects of SIPS

Although it is now well documented that ionizing radiation and chemotherapeutic agents induce a high degree of SIPS in *TP53* wild-type cancer cells, the pros and cons of this response in the context of therapeutic outcome have not yet been rigorously tested. Some authors consider SIPS to reflect a mechanism of elimination of cancer cells, analogous to apoptosis. However, as mentioned earlier, cells undergoing SIPS secrete myriad factors that can promote cell growth and invasiveness, as well as angiogenesis [13,112]. SIPS-associated secreted factors include pro-inflammatory cytokines, growth factors, matrix metalloproteinases, plasminogen activators, and fibronectin [112]. In addition, *in vitro* and xenograft studies with p53 wild-type human breast cancer cells have suggested the recovery of a proliferating cell population following an initial robust SIPS response post-irradiation ([85] and references therein).

There is also recent evidence suggesting that the crosstalk between SIPS and autophagy may contribute to tumor dormancy. Autophagy is a well-conserved lysosomal pathway used to degrade long-lived proteins and cytoplasmic organelles. Physiological levels of autophagy are essential for normal cellular homeostasis and play a prosurvival role, whereas excessive levels of autophagy promote autophagic cell death [113]. Autophagy and senescence are considered to be coupled responses that influence the capacity of the tumor cell to maintain a prolonged state of growth arrest (e.g., associated with SIPS) that can be succeeded by tumor regrowth and disease recurrence [114].

## 6. Responses Triggered by Moderate Doses of Ionizing Radiation in p53-Deficient Human Cells

In 1975, Taylor and associates [115] reported that cells derived from individuals with the genetic disorder ataxia telangiectasia (AT) exhibit greatly increased sensitivity to ionizing radiation when measured by the clonogenic survival assay. AT cells were subsequently reported to have a reduced ability to rejoin DSBs and to activate cell cycle checkpoints post-irradiation [116]. These observations are consistent with a model in which defects in DSB rejoining/checkpoints will lead to increased loss of clonogenic potential. Surprisingly, however, studies with Li-Fraumeni syndrome (LFS) fibroblast strains that harbor *TP53* mutations do not support this as a general model. Below we will first consider our observations with LFS strains and then discuss the radiation-induced responses in human solid tumor-derived cell lines that lack wild-type p53 function.

### 6.1. LFS Fibroblasts

**Figure 3:**
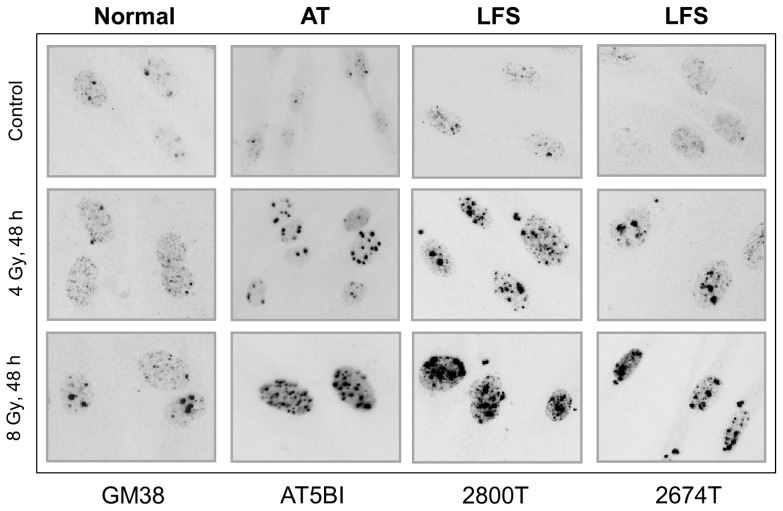
Immunofluorescence microscopy images showing γH2AX nuclear foci before (control) and 48 h after exposure of the indicated human fibroblast strains to ionizing radiation. The results are reproduced from [119] with permission.

The LFS fibroblast strains studied by us harbor either a germ line (codon 254; strains 2674T and 2675T) [117,118] or somatic (codon 234; strain 2800T) mutation [118] in one allele of the *TP53* gene. These mutations result in impairment of the cells’ p53 transcriptional transactivation capacity, as evidenced by their low degree of activation of a p53-regulated gene (*p21^WAF1^*) after exposure to DNA-damaging agents [66,119]. Using the neutral comet assay, we demonstrated that strains 2800T, 2674T and 2675T have a deficiency in the rejoining of DSBs similar to that of AT strains after exposure to an 8 Gy dose of γ radiation when compared to normal controls [119]. We also evaluated DSBs indirectly by the highly sensitive γH2AX foci fluorescence assay. As mentioned above, in this assay, DSBs that are detected by the cell are visualized as distinct nuclear foci. At 24 and 48 h after irradiation, we observed remarkably high levels of γH2AX foci in LFS and AT fibroblasts, but nearly undetectable (4 Gy) or low levels (8 Gy) of foci in normal fibroblasts [119] (also see Figure 3). Collectively, these observations with LFS strains are consistent with numerous reports demonstrating that wild-type p53 plays a role in DSB rejoining by interacting with various key players in homologous recombination (HR) and nonhomologous end joining (NHEJ), the two distinct but complimentary pathways for DSB repair (e.g., [27,120–122]).

The ability of 2800T fibroblasts to engage early cell cycle checkpoints has been evaluated indirectly by measuring DNA synthesis (tritiated-thymidine incorporation into genomic DNA) post-irradiation. As seen with other p53-deficient LFS fibroblasts [123,124], 2800T fibroblasts showed an abnormally high degree of DNA synthesis at ~10 h post-irradiation similar to AT fibroblasts [118], suggesting defective activation of early (presumably p53-dependent) checkpoints.

**Figure 4:**
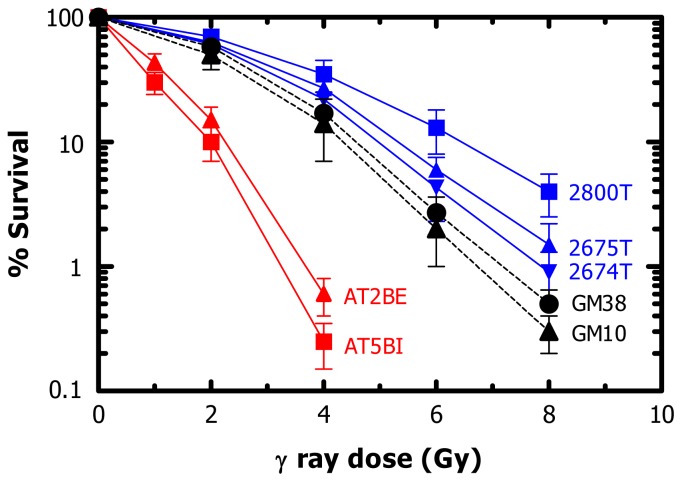
Clonogenic survival curves for human normal (GM10, GM38), ataxia telangiectasia (AT) (AT2BE, AT5BI) and Li-Fraumeni syndrome (LFS) (2800T, 2674T, 2675T) fibroblast strains after exposure to ionizing radiation. The results are reproduced from [119] with permission.

Persistence of DSBs at late times post-irradiation, coupled with the failure of cells to properly engage p53-dependent cell cycle checkpoints, would be expected to result in a high degree of lethality. While AT strains are highly radiosensitive in the clonogenic survival assay, LFS strains show increased *radioresistance* when compared to normal fibroblast strains [119,125] (also see Figure 4). Clearly apoptosis cannot account for the differential radiosensitivity of these strains because moderate doses of ionizing radiation (between 2 and 10 Gy) do not induce apoptosis in normal or AT fibroblasts [69,126].

It is important to note that the unexpected DNA damage response of these LFS strains is not limited to ionizing radiation. We [66,127] and others [128,129] have demonstrated that these strains are also deficient in aspects of nucleotide excision repair after exposure to UVC. The repair deficiency in these strains is similar to that seen in xeroderma pigmentosum (XP) complementation group E fibroblasts [66]. In the clonogenic assay, XPE fibroblasts show UV hypersensitivity, as expected, whereas LFS fibroblasts surprisingly show increased UV resistance when compared to normal fibroblasts [66].

Although the basis for the abnormal resistance of LFS strains to DNA-damaging agents remains elusive, these observations illustrate the complexity of the DNA damage response even in non-cancerous fibroblasts and call for a revision of the widely-cited model that merely integrates DNA repair, cell cycle checkpoints and apoptosis.

**Figure 5:**
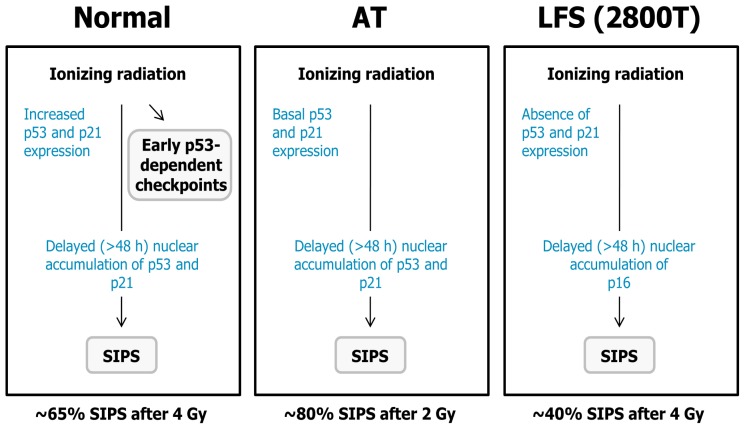
Schematic representation of the uncoupling of p53-dependent early (cell cycle checkpoints) and late (SIPS) responses induced by ionizing radiation in some genetic backgrounds. The observations for LFS were made using strain 2800T which does not exhibit wild-type p53 function at early passages [97].

How do LFS and AT fibroblasts lose their clonogenic potential post-irradiation? We used single-cell observation methods to address this question. To this end, we determined whether radiation exposure can trigger SIPS in these fibroblasts, and if so, whether this effect is associated with induction of p21 and/or p16^INK4A^ (hereafter p16). We found that moderate doses of ionizing radiation induced a high degree of SIPS in normal, LFS (2800T), and AT fibroblasts [97]. Induction of SIPS in p53 wild-type (normal and AT) fibroblasts correlated with sustained upregulation of p21 but not of p16, whereas in 2800T fibroblasts it correlated with induction of p16 but not of p21 [97]. In addition, we observed a relationship between the degree of radiosensitivity of these strains when measured by the clonogenic survival and SIPS assays. Exposure to a 4-Gy dose of γ radiation, for example, induced SIPS in ~65% and ~40% of the cells within cultures of normal (GM38) and radioresistant (2800T) strains, respectively; exposure to only a 2-Gy dose caused ~80% SIPS in cells within cultures of radiosensitive (AT2BE; AT5BI) strains [97]. Of note, the observation that ionizing radiation triggers SIPS in AT and LFS fibroblasts that do not engage the early cell cycle checkpoints post-irradiation indicates that these early (checkpoints) and late (SIPS) events are uncoupled in some genetic backgrounds (also see Figure 5).

### 6.2. Solid Tumor-Derived Cell Lines

Consistent with our findings with human fibroblast strains with differing *TP53* status, Wang and associates (supplementary data in [130]) reported that ionizing radiation triggers SIPS in non-small cell lung cancer cell lines with wild-type (e.g., A549) or mutant p53 (e.g., HCC44). Induction of SIPS was associated with expression of p21 and p16 in p53 wild-type and mutant cell lines, respectively. The authors concluded that in cancers that have mutations in either *p16^INK4A^* or *TP53*, the presence of the other wild-type gene product might be therapeutically exploited for induction of terminal growth arrest through SIPS.

**Figure 6:**
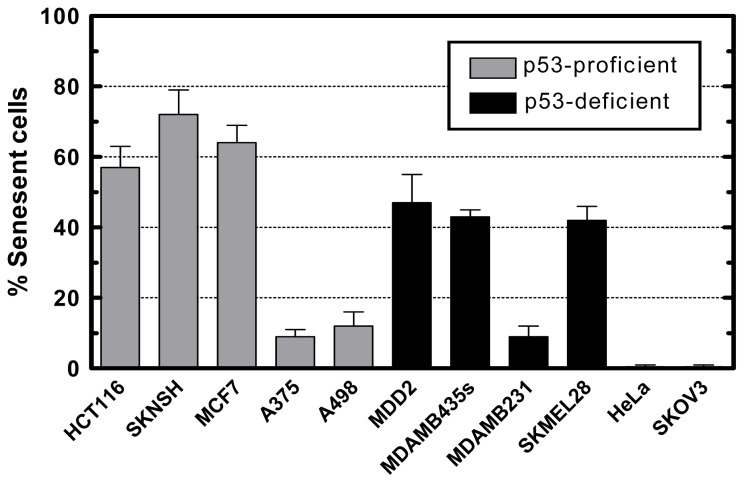
Induction of SIPS in the indicated human cancer cell lines measured at seven days after exposure to an 8-Gy dose of ^60^Co γ radiation. All values for irradiated cultures were corrected by subtracting those for sham-irradiated control cultures. Bars, SE. Cell lines marked “p53-proficient” express wild-type p53. Cell lines marked “p53-deficient” are either p53-null (SKOV3 and HeLa) or express mutant p53 (MDD2, MDA-MB-455s, MDA-MB-231, and SKMEL-28). The data for some cell lines are reproduced from our published work [67,131] with permission.

According to Wang *et al.* [130], only a small proportion of cells within p53-mutant cancer cell lines undergo SIPS post-irradiation. We have made a similar observation with some p53-mutated cancer cell lines used in our laboratory ([131] and Figure 6). In our studies, cultures were exposed to ^60^Co γ radiation (8 Gy), incubated for 7 days, and evaluated for features of SIPS (flattened and enlarged morphology and positive SA-β-gal staining). As shown in Figure 6, ionizing radiation induced SIPS in 10%–50% of cells within cultures of the p53-mutant cell lines MDA-MB-435s (breast carcinoma), MDA-MB-231 (breast carcinoma), MDD2 (breast carcinoma), and SK-MEL-28 (malignant melanoma), but did not induce SIPS in the p53-null cell lines HeLa (cervical carcinoma) and SKOV3 (ovarian carcinoma). Interestingly, some p53 wild-type cancer cell lines (e.g., A375 malignant melanoma and A498 renal carcinoma) also showed a low frequency (~10%) of SIPS after 8-Gy irradiation. This dose of ionizing radiation results in >95% loss of colony-forming ability in these cell lines [67,132].

What is the fate of cancer cells that fail to activate p53-dependent responses (e.g., early cell cycle checkpoints; SIPS) but remain viable after irradiation? Such cells often respond to ionizing radiation by duplicating their genome multiple times without undergoing cell division, a process called “endoreduplication”. This results in the development of “endopolyploid” giant cells containing a highly enlarged nucleus or multiple nuclei. The fate of endopolyploid giant cells is complex but fairly well characterized [4,133–146]. A proportion of giant cells first undergo a ploidy cycle, which is regulated by key mediators of mitosis (e.g., aurora B kinase), meiosis (e.g., MOS), and self-renewal (e.g., OCT4), ultimately giving rise to progeny with a near-diploid number of chromosomes (para-diploid) that regain proliferation potential (reviewed in [4]). The establishment of a para-diploid population from giant cells takes several weeks of incubation after the genotoxic insult. Although the frequency of giant cells that remain viable for long times post-irradiation might be low, the progeny of such cells may contribute to tumor regrowth. Indeed, Vitale *et al.* [138] reported that giant cells that develop in nocodazole-treated HCT116p53−/− cultures undergo multipolar and bipolar cell division, followed by a gradual decrease in genomic instability and return to para-diploidy within 2–3 weeks after treatment. Intriguingly, *in-vivo* studies in nude mice demonstrated that the isolated para-diploid progeny of HCT116p53−/− giant (tetraploid) cells caused the most rapid tumor growth when compared to parental and HCT116p53−/− cells [138].

Another mechanism of depolyploidization of polyploid/multinucleated giant cells is through a process called “neosis” which resembles the asexual cell division seen in budding yeast [133–135,139–144]. Neotic cell division associated with giant cells is fairly well characterized and has been documented for a variety of murine and human cell cultures, including solid tumor-derived cell lines [134,140,142,143]. Neosis-derived progeny that arise in response to genotoxic stress exhibit mitotic propagation and resistance to cancer therapeutic agents [133,134,139–141,144]. These findings provide further support for a role of giant cells and their para-diploid progeny in the recurrence of therapy-resistant malignancies.

As recently pointed out by Erenpreisa and Cragg [4], collectively, these intriguing discoveries “*provide an impetus for rethinking the biological nature of genotoxic resistance and self-renewal of tumor cells*.” Irrespective of their behavior in the conventional assays for measuring the various aspects of the DNA damage response (e.g., checkpoints; repair; proliferation; viability; apoptosis; clonogenic survival), a proportion of cells within a given tumor will enter a state of dormancy post-irradiation as a result of the development of polyploid/multinucleated giant cells. Such cells might sort out their highly unstable genome by different means involving neosis [134,145], ploidy cycle [4,146], and autophagy [135,145], ultimately contributing to disease recurrence [114,147] by giving rise to highly metastatic and therapy-resistant progeny.

## 7. Conclusions

Extensive research has been directed towards modulating p53 and other key players of the DNA damage surveillance network in an attempt to improve the outcome of conventional cancer therapies. This approach has met with limited success [148]. The impetus behind most of these studies has been the model proposed over a decade ago, suggesting that the principal role of the p53 pathway in determining cell fate following genotoxic stress is to either promote survival by activating cell cycle checkpoints and facilitating repair, or to induce apoptotic cell death. However, as discussed in this article, a large body of recent evidence has established that the primary response triggered by clinically relevant doses of ionizing radiation in human fibroblast stains and many solid tumor-derived cell lines is a sustained proliferation block, and not apoptosis. This proliferation block predominantly reflects SIPS in p53-proficient cultures, and the development of endopolyploid giant cells in p53-deficient cultures.

Unlike apoptotic and necrotic cells that are eliminated from the proliferating population, cells undergoing SIPS remain metabolically active and secrete factors with tumor-promoting activities [12,13,112]. In addition, studies with solid tumor-derived cells have suggested that a fraction of cells undergoing SIPS following genotoxic stress might escape the growth arrest and give rise to rapidly growing descendants [85,149]. Likewise, endopolyploid giant cells have the potential of giving rise to daughter cells that reenter the mitotic cell cycle and are often resistant to therapeutic agents [4,133–135]. Accordingly, there is growing interest in identifying agents (e.g., small molecule inhibitors) capable of triggering apoptosis of growth-arrested cancer cells, thereby abrogating their escape from standard therapies [59,147]. To this end, promising results have been reported with inhibitors of the mammalian target of rapamycin (mTOR) [150] and certain steroids [147,151].

Our understanding of the responses controlled by the p53–p21 axis has improved substantially, but is probably far from complete. The finding that ionizing radiation triggers sequential waves of p53 signaling which is primarily controlled by WIP1 is intriguing. The biological implications of this sequential wave response remain to be elucidated. Mathematical modeling has led to some speculations on the advantages of digital versus analogue induction of p53 signaling following DNA damage, but these speculations are centred on decision making by p53 in terms of “repair and survive” or “give up and die through apoptosis” (e.g., [81]). Numerous assumptions have been made to simplify such models, including p21 functioning only in the transient activation of the G1/S checkpoint and p53 existing in different forms (e.g., p53-helper which activates checkpoints through p21, p53-lurker which can be transformed into p53-killer, and p53-killer that triggers apoptosis). Although these speculations have not been verified experimentally, it is now well known that activation of p53 signaling by ionizing radiation does not trigger a significant degree of apoptosis in many human cell types, including solid tumor-derived cells [7,72].

Perhaps the cellular decision making between life and death is not entirely dependent on p53 as predicted by Oren a decade ago [10]. Our cells are programmed to withstand and reverse a “physiological” level of genetic changes, arising from endogenous and environmental sources. Under these conditions, the DNA damage-response machinery is rapidly activated and facilitates complete reversal of the genomic injury before the WIP1 effect is fully manifested. In response to therapeutic doses of ionizing radiation (2 Gy and above), however, the WIP1 effect precedes full repair, p21 blocks apoptosis, and p53 becomes up- and down-regulated sequentially until either the damage is reduced to a level compatible with cell survival or adaptation [152], or the cells undergo one of the available “death” options which is commonly p21-mediated SIPS but may also include apoptosis, necroptosis [153] and autophagy [154,155] which may contribute significantly to the response of some types of tumor cells to ionizing radiation.

Time will tell whether this interpretation is tenable. However, based on the current review article, in concert with recent reviews by us [7] and others [148,156], it is evident that a better understanding of the complexity of p53 signalling in general, and of the multiple functions of p21 in particular, is critical for designing effective p53/p21-based cancer therapies.

## Appendix

**Table A1:** Key review articles on the indicated topics.

Topics	References
DNA damage response	[2–4,7,11,12,14,57,70,157,158]
p53 structure	[7,159]
p53 regulation and signaling	[28,44,160,161]
p53-mediated responses through interaction with other proteins	[27]
MDM2/MDMX	[30,44,45,161–163]
WIP1	[33,42,161]
Multiple functions of p21 in the DNA damage response	[7,156,164]
Mechanisms of radiation toxicity	[11,12,14,70]
p21-dependent SIPS post-irradiation	[7,12,57]
p16-dependent SIPS post-irradiation	[131]
p53-based cancer therapies	[7,148]
